# Early treatment with ambrisentan of mildly elevated mean pulmonary arterial pressure associated with systemic sclerosis: a randomized, controlled, double-blind, parallel group study (EDITA study)

**DOI:** 10.1186/s13075-019-1981-0

**Published:** 2019-10-26

**Authors:** Zixuan Pan, Alberto M. Marra, Nicola Benjamin, Christina A. Eichstaedt, Norbert Blank, Eduardo Bossone, Antonio Cittadini, Gerry Coghlan, Christopher P. Denton, Oliver Distler, Benjamin Egenlauf, Christine Fischer, Satenik Harutyunova, Panagiota Xanthouli, Hanns-Martin Lorenz, Ekkehard Grünig

**Affiliations:** 1 Centre for Pulmonary Hypertension, Thoraxklinik at Heidelberg University Hospital, Röntgenstraße 1, 69126 Heidelberg, Germany; 2 Translational Lung Research Center Heidelberg (TLRC), Member of the German Center for Lung Research (DZL), Heidelberg, Germany; 3 IRCCS SDN Research Institute, Naples, Italy; 4 Department of Human Genetics, University of Heidelberg, Heidelberg, Germany; 5 Department of Rheumatology, University Hospital Heidelberg, Heidelberg, Germany; 6 Division of Cardiology, U.O.C. Rehabilitazione Cardiovascolare, A Cardarelli, Naples, Italy; 7 Department of Translational Medical Sciences, University Federico II of Naples, Naples, Italy; 8 Cardiology Department, Royal Free Hospital, London, UK; 9 Centre of Rheumatology, Royal Free Hospital, London, UK; 10 Department of Rheumatology, University Hospital Zurich, Zurich, Switzerland

**Keywords:** Mildly elevated mPAP, Borderline pulmonary hypertension, Exercise PH, Ambrisentan, Treatment, Placebo-controlled

## Abstract

**Objective:**

The objective of this randomized, placebo-controlled, double-blind, parallel group, trial was to assess the effect of ambrisentan on mean pulmonary arterial pressure (mPAP) in patients with systemic sclerosis (SSc) and mildly elevated pulmonary hypertension (PH).

**Methods:**

Thirty-eight SSc patients with mildly elevated mPAP at rest between 21 and 24 mmHg and/or > 30 mmHg during low-dose exercise were randomly assigned to treatment with either ambrisentan 5–10 mg/day or placebo. Right heart catheterization and further clinical parameters were assessed at baseline and after 6 months. The primary endpoint was the difference of mPAP change at rest between groups.

**Results:**

After 6 months, the two groups did not differ in the primary endpoint (ambrisentan mPAP − 1 ± 6.4 mmHg vs. placebo − 0.73 ± 3.59 mmHg at rest, *p* = 0.884). However, three patients from the placebo group but none of the ambrisentan group progressed to SSc-associated pulmonary arterial hypertension. Furthermore, ambrisentan treatment showed significant improvements in the secondary endpoints cardiac index (CI) and pulmonary vascular resistance (PVR) at rest (CI 0.36 ± 0.66 l/min/m^2^ vs. − 0.31 ± 0.71 l/min/m^2^, *p* = 0.010; PVR − 0.70 ± 0.78 WU vs. 0.01 ± 0.71 WU, *p* = 0.012) and during exercise (CI 0.7 ± 0.81 l/min/m^2^ vs. − 0.45 ± 1.36 l/min/m^2^, *p* = 0.015; PVR − 0.84 ± 0.48 WU vs. − 0.0032 ± 0.34 WU, *p* < 0.0001).

**Conclusion:**

This is the first randomized, double-blind, placebo-controlled study testing the effect of ambrisentan in patients with mildly elevated mPAP and/or exercise PH. The primary endpoint change in mPAP did only tendentially improve in the ambrisentan group, but the significant improvement of other hemodynamic parameters points to a possible benefit of ambrisentan and will be helpful to design future trials.

**Trial registration:**

www.ClinicalTrials.gov, unique identifier NCT: NCT02290613, registered 14^th^ of November 2014.

## Background

Pulmonary hypertension (PH) is a manifestation of systemic sclerosis (SSc) [1] which dramatically impairs patients’ prognosis [2]. In the American REVEAL registry, patients with SSc-associated-pulmonary arterial hypertension (PAH) (SSc-APAH) had the worst 1-, 2-, and 3-year survival rates of 78, 58, and 47%, respectively [3]. If left untreated, SSc-APAH patients have a median survival rate of 1 year after diagnosis [4]. Mildly elevated mean pulmonary arterial pressure (mPAP) of 21–24 mmHg has been shown to be associated with impaired exercise capacity and poorer outcomes when compared with individuals with mPAP within the normal range [5–7, 38]. A recently published post hoc analysis of the DETECT study showed that SSc patients with mPAP of 21–24 mmHg were also more prone to develop manifest PH after 2.95 ± 0.7 years follow-up (chi-square *p* value 0.0226) than SSc patients with mPAP of ≤ 20 mmHg [8]. In a recent study, patients with SSc and mildly elevated mPAP presented with impaired pulmonary arterial compliance and reduced increase of cardiac index (CI) assessed by right heart catheterization (RHC) during exercise [9]. During the 6^th^ World Symposium on Pulmonary Hypertension, a new definition of PH has been proposed, lowering the cutoff for mPAP from ≥ 25 to > 20 mmHg and including the criterion of a pulmonary vascular resistance (PVR) > 3 Wood Units for all forms of precapillary PH [10]. This new definition may allow an earlier identification of PH in SSc patients.

Ambrisentan is an endothelin receptor antagonist with endothelin receptor A selectivity, which was developed for the targeted treatment of PAH. Ambrisentan was evaluated in two large phase III randomized controlled trials in ARIES (ambrisentan in pulmonary arterial hypertension, randomized, double-blind, placebo-controlled, multicenter, efficacy study)-1 and ARIES-2 for the treatment of PAH [11]. In both studies, ambrisentan has demonstrated beneficial effects on PAH symptoms, exercise capacity, hemodynamics, and time to clinical worsening, defined as occurrence of death, lung transplantation, atrial septostomy, and study withdrawal because of addition of other PAH medication or “early escape” criteria (the occurrence of two of the following: > 20% decrease of a 6-min walking distance (6MWD), increase in the World Health Organization functional class (WHO-FC), worsening right ventricular function, hepatic or renal failure, systolic blood pressure < 85 mmHg). In PAH patients, mPAP improved about 15% due to ambrisentan [12]. Due to its beneficial effects on the development of digital ulcerations in patients with SSc [13, 14], the endothelin receptor antagonist bosentan has been approved for the treatment of digital ulcers.

Up to now, apart from two small, uncontrolled open-label reports [15, 16], data regarding the treatment of mildly elevated mPAP and/or exercise PH is lacking. As an early recognition and management of PAH in SSc grants a significant survival benefit [17], there is a high need of randomized, controlled studies investigating the effect of PAH-targeted treatment in SSc patients with mildly elevated mPAP and/or exercise PH. As ambrisentan seems to have a better safety profile than the endothelin receptor antagonist bosentan with regard to hepatic safety and drug-drug interactions [18], we aimed to investigate the effect of ambrisentan on patients with SSc and mildly elevated mPAP and/or exercise PH.

The aim of this study was therefore to investigate, whether an early treatment in patients with mildly elevated mPAP and/or exercise PH may prevent these patients from worsening of hemodynamics. Mean PAP has been chosen as a primary endpoint in order to detect the progression to manifest PH.

## Material and methods

### Study subjects

Patients affected by diffuse cutaneous SSc (dcSSc) and limited cutaneous SSc (lcSSc) [19] referred to our center for the purpose of PH screening which was performed according to a modified DETECT algorithm were enrolled into the study [20]. All SSc patients received a complete non-invasive clinical assessment including echocardiography at rest and right heart catheterization at rest and during exercise. The patients were enrolled in this study if they had either (1) resting mPAP 21–24 mmHg, pulmonary arterial wedge pressure (PAWP) < 15 mmHg, transpulmonary gradient (TPG = mPAP-PAWP) > 11 mmHg, or (2) exercise-induced elevated mPAP values > 30 mmHg, PAWP < 18 mmHg, and TPG > 15 mmHg which occurred at low workloads (cardiac output (CO) < 10 l/min) [21] without significant left heart or severe lung disease. Inclusion of patients was based on pulmonary pressures and not on PVR, as this criterion [10] was not yet implemented during the conduct of the study. Left heart disease was assessed by clinical examinations including electrocardiogram (ECG), echocardiography, stress echocardiography, stress ECG, and laboratory testing of the N-terminal fraction of the pro-brain natriuretic peptide and troponin T (NTproBNP, TnT ≥ 14). In the case of suspected coronary artery disease or any other left heart disease in patients with elevated wedge pressures, patients were referred for left heart catheterization. Lung diseases were assessed by lung function tests, by chest X-ray, and if clinically indicated by high-resolution computed tomography (HRCT). Lung involvement of SSc was considered significant when forced vital capacity (FVC) was < 60%, or HRCT showed severe fibrosis, or when FVC was 60–70% and HRCT showed moderate-severe fibrosis or was “not available,” as previously described [20]. Diagnosis of SSc was confirmed by experienced rheumatologists (HML, NoB) according to the standard criteria of the American Rheumatism Association [22] with a SSc disease duration from first non-Raynaud symptoms > 3 years. Manifest PH/PAH was diagnosed according to the current ERS/ESC guidelines [23].

A written informed consent was obtained from all patients in accordance with the Declaration of Helsinki. The approval of the ethics committee of the Medical Faculty Heidelberg was obtained (AFmo-407/2014); the trial was registered on www.ClinicalTrials.gov (unique identifier NCT: NCT02290613) and in the European Clinical Trial Register (EudraCT Number: 2014-001882-28).

### Study design

The EDITA study was a single-center (PH Center, Thoraxklinik at Heidelberg University Hospital, Heidelberg, Germany) investigator-initiated trial using a prospective, randomized, double-blind (patient and investigator), parallel group, placebo-controlled, phase IIA clinical study design. Patients were randomized 1:1 to either ambrisentan or placebo by simple randomization. Medication was prepared at the Department of Medical Pharmacy, University of Heidelberg, according to the allocation order and labeled with the patient numbers. Treatment allocation was concealed until database closure. Ambrisentan treatment was started at a dose of 5 mg/day and uptitrated to 10 mg/day after 1–4 weeks according to tolerability. Placebo tablets with the same color and shape as ambrisentan were provided by GlaxoSmithKline. Patients were assessed at baseline, after 3 and 6 months including medical history, demographics, concomitant medication, physical examination, WHO-FC, vital signs, SSc characteristics, 12-lead ECG, 6MWD including Borg dyspnea score, 2-dimensional-echocardiography at rest, clinical laboratory including pregnancy test, lung function test with blood gas analysis, and monitoring of adverse events. Modified Rodnan skin score (mRSS), echocardiography, RHC with continuous 3-lead ECG at rest and during exercise, and quality of life (QoL) questionnaire (short form health survey SF-36) were performed at baseline and after 6 months only. An appropriate contraception throughout the study was required for women with child-bearing potential.

### Endpoints

The primary endpoint was the change from baseline to 6 months in resting mPAP with ambrisentan compared to change with placebo.

Secondary endpoints included changes from baseline to 6 months in further invasively measured hemodynamic parameters at rest and during exercise (RHC: right atrial pressure, PVR, CO, cardiac index (CI), PAWP, venous oxygen saturation (SvO_2_)), WHO-FC, mRSS and symptoms of SSc (presence of (digital) ulcers, calcinosis, dysphagia, telangiectasia, Raynaud phenomenon, and joint pain), 6MWD, QoL (SF-36), lung function tests, right heart dimensions and function assessed with transthoracic echocardiography, and NTproBNP. Measures of disease-related progression (adverse events, hospitalization, and initiation of PAH treatment) and the assessment of tolerability and safety were assessed throughout the study and during the safety follow-up period.

### Study procedures

The hemodynamic values were obtained by RHC. The RHC was performed in a standardized way in a supine position using the transjugular access with a triple-lumen 7F-Swan-Ganz thermodilution catheter at rest and during exercise as previously described [9]. Resting two-dimensional transthoracic echocardiography Doppler examinations were performed by experienced cardiac sonographers (EG, AMM, SH) with commercially available equipment (Vivid 9, GE Healthcare, Milwaukee, WI) according to a standardized protocol as described previously [24].

### Statistical analysis

Statistical analyses were conducted by two statisticians (CF, NB). Data are described as mean ± standard deviation (SD) and 95% confidence interval or number and %, respectively.

Based on data from the literature [16], we calculated that 15 patients (18 including a 20% dropout rate) would have 90% power to detect a placebo-corrected mean difference of mPAP (baseline to 6 months) of 3 ± 2.5 mmHg (equal standard deviation) at a two-sided significance level of 0.05. The primary efficacy analysis was performed on data from the intention-to-treat population (all patients who received randomization) by a *t*-test with unequal variances (Welch tests) since the assumption for a covariance analysis was not fulfilled.

Secondary hemodynamic endpoints were tested with *t*-tests for unequal variances. Safety was analyzed descriptively. Adverse events during the study period included all adverse events that started or worsened at the time of administration of the first dose of study drug until the last visit (6 months).

Total pulmonary resistance (TPR) was calculated as mPAP-CO slope using the formula TPR = mPAP at peak exercise/CO at peak exercise [21]. All tests were two-tailed, and *p* values < 0.05 were considered as statistically significant. Secondary endpoints were tested exploratory. As sensitivity analysis, changes in WHO-FC, 6-min walking test, QoL, and skin fibrosis were analyzed in patients with dcSSc only. All analyses were performed with SPSS V25 (SPSS Statistics V25, IBM Corporation, Somers, NY) and JMP14 (SAS Institute, Cary, NC).

## Results

**Fig. 1:**
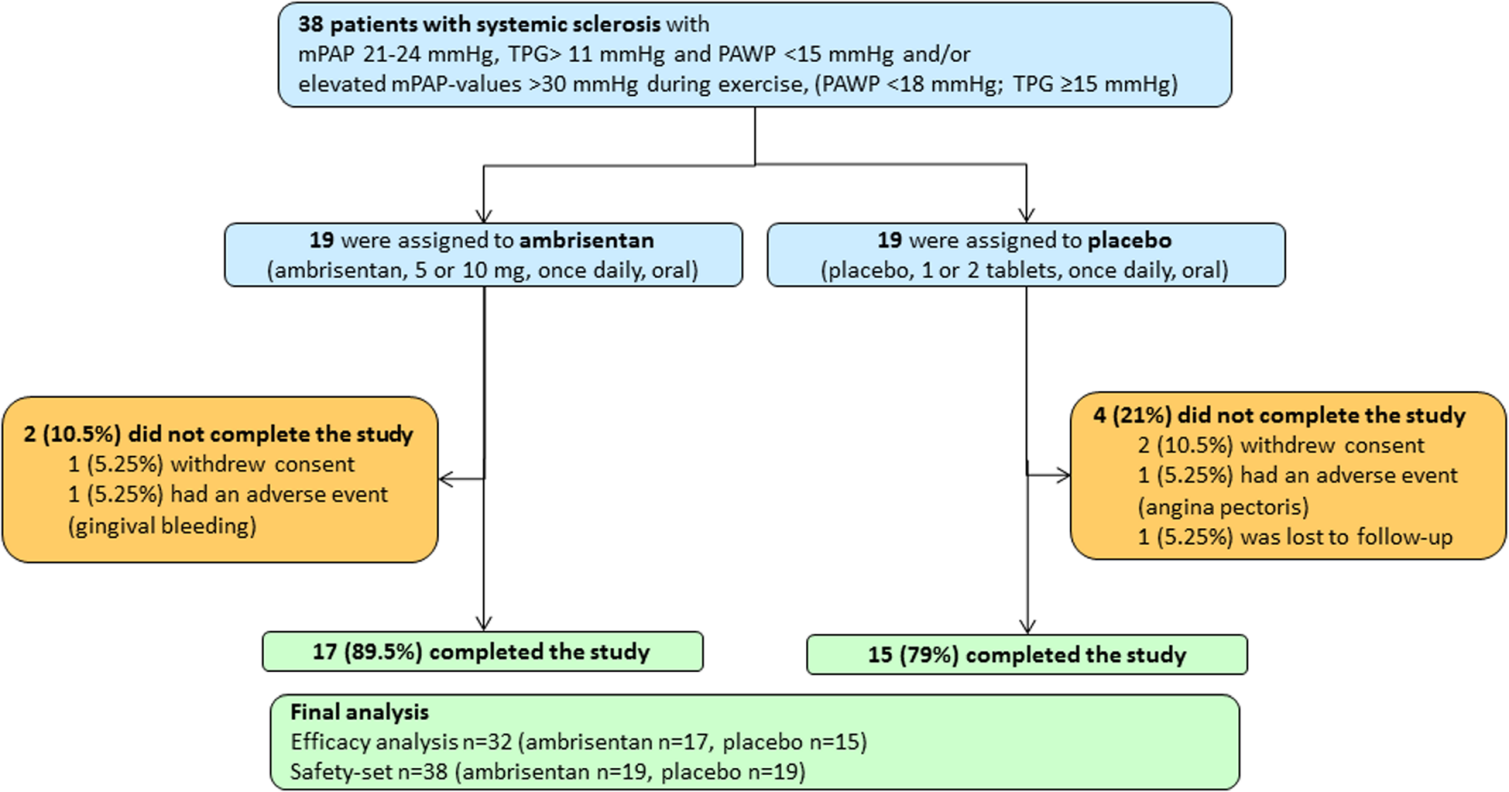
Study flowchart. A total of 38 patients were enrolled into our study and randomly assigned to be treated with ambrisentan 5 or 10 mg/day or to receive placebo. After 6 months, 32 patients completed the study, 17 in the ambrisentan group, and 15 in the placebo group. Among the 6 dropout patients, 1 in the ambrisentan group and 2 in the placebo group withdrew their written informed consents. One in each group quit because of adverse events, one in the ambrisentan group for gingival bleeding, and one in the placebo group for angina pectoris. One patient in the placebo group was lost to follow-up

In this study, 38 SSc patients were randomly assigned to receive placebo or ambrisentan (5–10 mg/day) in the EDITA study from December 2014 until April 2017. Of these, 32 patients completed the study (15 in the placebo group and 17 in the ambrisentan group, Fig. 1): 78.9% female, mean age 56.8 ± 11.0 years, 39.5% dcSSc, 60.5% lcSSc. All of the 38 patients had already symptoms such as shortness of breath during exercise. Baseline parameters were well balanced between the two groups (Table 1), except for SSc subtype and duration of SSc.

**Table 1:** Demographic and clinical characteristics of the study population

Parameter [unit]	Placebo (*N* = 19)			Ambrisentan (*N* = 19)				Total (*N* = 38)		
	Mean ± SD	95% CI	*n*	Mean ± SD	95% CI	*n*	95% CI of difference	Mean ± SD	95% CI	*n*
Female sex no. [%]	14 (73.7)			16 (84.2)				30 (78.9)		
Age [years]	54.89 ± 11.23	(49.48 to 60.31)		58.79 ± 10.75	(53.61 to 63.97)		(− 3.33 to 11.12)	56.84 ± 11.02	(53.22 to 60.46)	
Height [cm]	166.21 ± 10.19	(161.30 to 171.12)		166.05 ± 5.59	(163.36 to 168.75)		(− 5.62 to 5.31)	166.13 ± 8.11	(163.47 to 168.80)	
Weight [kg]	74.81 ± 17.25	(66.48 to 83.10)		71.45 ± 15.83	(64.03 to 79.22)		(− 14.03 to 7.70)	73.13 ± 16.42	(67.83 to 78.59)	
Systolic blood pressure [mmHg]	121.32 ± 14.99	(114.09 to 128.54)		116.32 ± 11.53	(110.76 to 121.87)		(− 13.80 to 3.80)	118.82 ± 13.43	(114.40 to 123.23)	
Diastolic blood pressure [mmHg]	73.42 ± 9.14	(69.02 to 77.82)		73.68 ± 8.95	(69.37 to 78.00)		(− 5.69 to 6.21)	73.55 ± 8.92	(70.62 to 76.49)	
HR [beats/min]	77.58 ± 9.85	(72.83 to 82.33)		73.05 ± 11.12	(67.69 to 78.41)		(− 11.44 to 2.39)	75.32 ± 10.61	(72.83 to 78.81)	
WHO-FC no. [%]										
II	15 (78.9)			17 (89.5)				32 (84.2)		
III	4 (21.1)			2 (10.5)				6 (15.8)		
Hemodynamic characteristic according to inclusion criteria										
mPAP rest 21–24 mmHg, mPAP exercise > 30 mmHg	12 (63.2)			10 (52.6)				22 (57.9)		
mPAP rest 21–24 mmHg, mPAP exercise ≤ 30 mmHg	3 (15.8)			1 (5.3)				4 (10.5)		
mPAP rest < 21 mmHg, mPAP exercise > 30 mmHg	4 (21.0)			8 (42.1)				2 (31.6)		
mRSS [points]	11.47 ± 5.22	(8.72 to 14.23)		11.47 ± 5.71	(8.96 to 13.99)		(− 3.60 to 3.60)	11.47 ± 5.40	(9.70 to 13.25)	
SSc subgroup, no. (%)										
Diffuse	11 (57.9)			4 (21.1)				15 (39.5)		
Limited	8 (42.1)			15 (78.9)				23 (60.5)		
SSc disease duration [years]	13.28 ± 10.85	(8.05 to 18.51)		6.63 ± 4.80	(4.32 to 8.96)		(− 12.17 to 1.12)	9.96 ± 8.93	(7.02 to 12.90)	
Hemodynamics at rest										
CVP [mmHg]	5.63 ± 2.97	(4.20 to 7.06)		6.11 ± 2.94	(4.69 to 7.52)		(− 1.47 to 2.41)	5.87 ± 2.92	(4.90 to 6.82)	
mPAP [mmHg]	21.32 ± 2.43	(20.15 to22.49)		19.84 ± 3.58	(18.12 to 21.57)		(− 3.49 to 0.54)	20.58 ± 3.11	(19.56 to 21.60)	
PAWP [mmHg]	9.58 ± 2.97	(8.15 to 11.01)		9.42 ± 2.32	(8.30 to 10.54)		(− 1.91 to 1.59)	9.50 ± 2.63	(8.64 to 10.37)	
CO [l/min]	5.66 ± 1.48	(4.95 to 6.37)		5.04 ± 1.26	(4.43 to 5.65)		(− 1.51 to 0.29)	5.35 ± 1.39	(4.89 to 5.81)	
CI [l/min/m^2^]	3.20 ± 0.85	(2.79 to 3.61)		2.84 ± 0.64	(2.53 to 3.14)		(− 0.86 to 0.13)	3.02 ± 0.76	(2.77 to 3.27)	
SvO_2_ [%]	73.13 ± 4.60	(70.67 to 75.58)	(16)	73.91 ± 8.58	(69.50 to 78.32)	(17)	(− 4.15 to 5.71)	73.53 ± 6.85	(71.10 to 75.96)	(33)
PVR [WU]	2.09 ± 0.61	(1.80 to 2.39)		2.22 ± 0.93	(1.77 to 2.67)		(− 0.39 to 0.65)	2.16 ± 0.78	(1.90 to 2.41)	
Hemodynamics at peak exercise										
mPAP [mmHg]	36.94 ± 6.08	(33.82 to 40.07)	(17)	37.78 ± 3.61	(35.98 to 39.57)	(18)	(− 2.58 to 2.25)	37.37 ± 4.91	(35.69 to 39.06)	(35)
PAWP [mmHg]	15.24 ± 5.43	(12.45 to 18.03)	(17)	16.78 ± 6.42	(13.58 to 19.97)	(18)	(− 2.56 to 5.64)	16.03 ± 5.92	(13.99 to 18.06)	(35)
CO [l/min]	11.03 ± 3.62	(9.17 to 12.89)	(17)	9.67 ± 2.67	(8.34 to 10.99)	(18)	(− 3.54 to 0.81)	10.33 ± 3.19	(9.23 to 11.43)	(35)
CI [l/min/m^2^]	6.04 ± 1.79	(5.12 to 6.96)	(17)	5.41 ± 1.32	(4.75 to 6.06)	(18)	(− 1.71 to 0.45)	5.71 ± 1.58	(5.17 to 6.25)	(35)
SvO_2_ [%]	38.27 ± 6.10	(34.89 to 41.64)	(15)	42.77 ± 11.56	(36.36 to 49.17)	(15)	(− 2.42 to 11.42)	40.52 ± 9.37	(37.02 to 44.02)	(30)
Workload [Watt]	75.00 ± 34.23	(57.40 to 92.60)	(17)	75.00 ± 27.12	(61.52 to 88.48)	(18)	(− 21.18 to 21.17)	75.00 ± 30.32	(65.59 to 85.41)	(35)
HR max [/min]	117.65 ± 21.37	(106.66 to 128.63)	(17)	111.39 ± 23.06	(99.92 to 122.86)	(18)	(− 21.57 to 9.06)	114.43 ± 22.15	(106.82 to 122.04)	(35)
PVR [WU]	2.07 ± 0.61	(1.76 to 2.38)	(17)	2.29 ± 0.85	(1.87 to 2.71)	(18)	(− 0.29 to 0.73)	2.18 ± 0.74	(1.93 to 2.44)	(35)
TPR [mmHg min l^− 1^]	3.65 ± 1.18	(3.04 to 4.26)	(17)	4.28 ± 1.65	(3.46 to 5.10)	(18)	(− 0.36 to 1.62)	3.97 ± 1.46	(3.47 to 4.47)	(35)
6MWD										
6MWD [m]	448.11 ± 82.64	(408.28 to 487.93)		470.21 ± 77.03	(433.08 to 407.34)		(− 30.46 to 74.67)	459.16 ± 79.59	(433.00 to 485.32)	
Borg dyspnea score	2.93 ± 1.97	(1.98 to 3.88)		2.41 ± 1.32	(1.77 to 3.04)		(− 1.63 to 0.58)	2.67 ± 1.68	(2.11 to 3.22)	
SaO_2_ after 6MWD [%]	91.87 ± 4.63	(89.30 to 94.43)	(15)	91.71 ± 4.79	(89.24 to 94.17)	(17)	(− 3.57 to 3.25)	91.78 ± 4.64	(90.10 to 93.45)	(32)
HR after 6MWD [/min]	99 ± 21.27	(88.07 to 109.93)	(17)	107 ± 19.64	(96.90 to 117.10)	(17)	(− 6.30 to 22.30)	103 ± 20.56	(95.82 to 110.17)	(34)
Echocardiography at rest										
Estimated sPAP [mmHg]	29.21 ± 5.16	(25.25 to 31.17)		28.58 ± 6.57	(26.04 to 31.43)		(− 4.52 to 3.26)	28.89 ± 5.83	(26.98 to 30.81)	
RA area [cm^2^]	12.05 ± 4.24	(10.73 to 13.69)		11.68 ± 3.28	(10.45 to 13.66)		(− 2.86 to 2.13)	11.87 ± 3.74	(10.64 to 13.10)	
RV area [cm^2^]	14.89 ± 4.56	(12.97 to 17.03)		14.13 ± 4.74	(12.65 to 16.82)		(− 3.82 to 2.30)	14.51 ± 4.60	(13.00–16.03)	
TAPSE [cm]	2.50 ± 0.51	(2.23 to 2.71)		2.41 ± 0.33	(2.28 to 2.70)		(− 0.37 to 0.20)	16,834 ± 0.43	(2.31 to 2.60)	
Lung function										
FVC [%]	69.17 ± 13.72	(62.56 to 75.78)		70.51 ± 17.61	(62.02 to 79.00)		(− 6.37 to 1.84)	69.84 ± 15.58	(64.72 to 74.96)	
FEV1 [L]	2.40 ± 0.77	(2.03 to 2.77)		2.26 ± 0.63	(1.96 to 2.57)		(− 0.60 to 0.33)	2.33 ± 0.70	(2.10 to 2.56)	
FEV1% VC max [%]	79.31 ± 6.98	(75.95 to 82.68)		81.44 ± 15.38	(74.03 to 88.85)		(− 5.73 to 9.99)	80.38 ± 11.83	(76.49 to 84.27)	
PEF [l/s]	5.46 ± 2.32	(4.34 to 6.58)		5.17 ± 1.72	(4.34 to 6.00)		(− 1.63 to 1.06)	5.31 ± 2.02	(4.65 to 5.98)	
TLC [l]	4.96 ± 1.07	(4.44 to 5.48)		5.08 ± 1.25	(4.48 to 5.69)		(− 0.64 to 0.89)	5.02 ± 1.15	(4.64 to 5.40)	
Residual volume [l]	1.94 ± 0.55	(1.68 to 2.21)		2.18 ± 0.70	(1.84 to 2.52)		(− 0.18 to 0.65)	2.06 ± 0.63	(1.85 to 2.27)	
DLCO [mmol/min/kPa]	5.28 ± 1.52	(4.55 to 6.02)		5.03 ± 1.33	(4.38 to 5.67)		(− 1.20 to 0.68)	5.16 ± 1.42	(4.69 to 5.62)	
DLCO % predicted	84.73 ± 2.61	(83.47 to 85.99)		84.16 ± 1.89	(83.25 to 85.08)		(− 2.07 to 0.93)	84.45 ± 2.27	(83.70 to 85.19)	
SaO_2_ [%]	96.13 ± 1.80	(95.26 to 97.00)		96.82 ± 0.77	(96.45 to 97.17)		(− 0.23 to 1.60)	96.47 ± 1.41	(96.01 to 96.94)	
PaO_2_ [mmHg]	78.78 ± 9.42	(74.24 to 83.32)		81.22 ± 5.91	(78.37 to 84.06)		(− 2.74 to 7.61)	80.00 ± 7.85	(77.42 to 82.58)	
PaCO_2_ [mmHg]	39.01 ± 3.63	(37.25 to 40.76)		37.23 ± 2.57	(35.99 to 38.47)		(− 3.85 to 0.29)	38.12 ± 3.23	(37.05 to 39.18)	
Laboratory										
Hemoglobin [g/dl]	13.54 ± 1.26	(12.93 to 14.14)		13.62 ± 1.13	(13.07 to 14.16)		(− 0.71 to 0.87)	13.58 ± 1.18	(13.19 to 13.96)	
Hematocrit [l/l]	0.42 ± 0.03	(0.40 to 0.43)		0.41 ± 0.03	(0.40 to 0.43)		(− 0.03 to 0.02)	0.41 ± 0.03	(0.40 to 0.42)	
Platelet [100/nl]	2.53 ± 0.94	(2.08 to 2.98)		2.62 ± 0.63	(2.31 to 2.92)		(− 0.44 to 0.61)	2.58 ± 0.79	(2.32 to 2.83)	
Creatinine [mg/dl]	0.83 ± 0.16	(0.75 to 0.91)		0.86 ± 0.12	(0.80 to 0.92)		(− 0.07 to 0.13)	0.85 ± 0.14	(0.80 to 0.89)	
Potassium [mmol/l]	4.05 ± 0.38	(3.86 to 4.23)		4.20 ± 0.48	(3.97 to 4.43)		(− 0.13 to 0.44)	4.12 ± 0.43	(3.98 to 4.27)	
AST [U/l]	24.11 ± 21.78	(13.61 to 34.60)		19.79 ± 7.06	(16.39 to 23.19)		(− 14.97 to 6.34)	21.95 ± 16.12	(16.65 to 27.25)	
ALT [U/l]	29.68 ± 24.93	(17.67 to 41.70)		24.68 ± 9.85	(19.93 to 29.43)		(− 17.47 to 7.47)	27.18 ± 18.87	(20.98 to 33.39)	
LDH [U/l]	197.63 ± 54.74	(171.25 to 224.01)		197.53 ± 35.59	(180.37 to 214.68)		(− 30.48 to 30.27)	197.58 ± 45.54	(182.61 to 212.55)	
CRP [mg/l]	5.18 ± 5.17	(2.69 to 7.68)		5.28 ± 11.60	(− 0.48 to 11.05)	(18)	(− 5.84 to 6.04)	5.23 ± 8.77	(2.30 to 8.16)	(37)
NTproBNP [pg/ml]	123.42 ± 142.96	(54.52 to 192.32)		267.83 ± 303.11	(117.10 to 418.57)	(18)	(− 12.38 to 301.20)	193.68 ± 242.81	(112.71 to 274.63)	(37)
Quality of life SF-36										
Physical functioning	50.26 ± 25.95	(37.75 to 62.77)		64.21 ± 25.83	(51.67 to 76.66)		(− 3.09 to 30.99)	57.24 ± 26.50	(48.52 to 65.95)	
Physical role functioning	35.53 ± 40.24	(16.13 to 54.92)		51.32 ± 41.23	(31.45 to 79.19)		(− 11.01 to 42.59)	43.42 ± 40.97	(29.96 to 56.89)	
Bodily pain	49.79 ± 28.96	(35.83 to 63.75)		62.00 ± 29.16	(47.95 to 76.05)		(− 6.91 to 31.33)	55.89 ± 29.33	(46.26 to 65.53)	
General health perceptions	41.74 ± 13.07	(35.44 to 48.04)		54.42 ± 19.47	(45.03 to 63.81)		(1.72 to 23.65)	48.08 ± 17.58	(42.30 to 53.86)	
Vitality	42.89 ± 19.32	(33.58 to 52.20)		50.53 ± 20.94	(40.03 to 60.62)		(− 5.62 to 20.89)	46.71 ± 20.24	(40.06 to 53.37)	
Social role functioning	58.05 ± 25.99	(45.53 to 70.58)		74.47 ± 24.45	(62.69 to 86.26)		(− 0.18 to 33.03)	66.26 ± 26.24	(57.64 to 74.89)	
Emotional role functioning	49.16 ± 46.33	(26.83 to 71.49)		63.16 ± 47.02	(40.50 to 85.82)		(− 16.71 to 44.71)	56.16 ± 46.58	(40.85 to 71.47)	
Mental health	59.58 ± 18.85	(50.49 to 68.66)		64.42 ± 18.03	(55.73 to 73.11)		(− 7.30 to 16.98)	62.00 ± 18.36	(55.97 to 68.04)	
Physical health score	43.89 ± 21.26	(33.65 to 54.14)		56.47 ± 23.87	(44.97 to 67.98)		(− 2.29 to 27.45)	50.18 ± 23.19	(42.56 to 57.81)	
Mental health score	50.26 ± 20.70	(40.29 to 60.24)		61.42 ± 21.45	(51.08 to 71.76)		(− 2.71 to 25.03)	55.84 ± 21.55	(48.76 to 62.92)	

*SD* standard deviation, *HR* heart rate, *mRSS* modified Rodnan Skin Score, *WHO-FC* World Health Organization functional class, *CVP* central venous pressure, *mPAP* mean pulmonary arterial pressure, *PAWP* pulmonary capillary wedge pressure, *CO* cardiac output, *CI* cardiac index, *SvO*_*2*_ venous oxygen saturation, *PVR* pulmonary vascular resistance, *WU* Wood Units, *sPAP* systolic pulmonary arterial pressure, *RA* right atrial, *RV* right ventricular, *TAPSE* tricuspid annular plane systolic excursion, *FVC* forced vital capacity, *FEV1* forced expiratory volume in first second, *VC* vital capacity, *PEF* peak expiratory flow, *TLC* total lung capacity, *DLCO* diffusing capacity of the lung for carbon monoxide, *SaO*_*2*_ oxygen saturation, *PaO*_*2*_ partial pressure of oxygen, *PaCO*_*2*_ partial pressure of carbon dioxide, *AST* aspartate-aminotransferase, *ALT* alanine-aminotransferase, *LDH* lactate dehydrogenase, *CRP* C-reactive protein

In case of missing data, sample sizes are given in brackets

### Hemodynamics at rest and during exercise

**Fig. 2:**
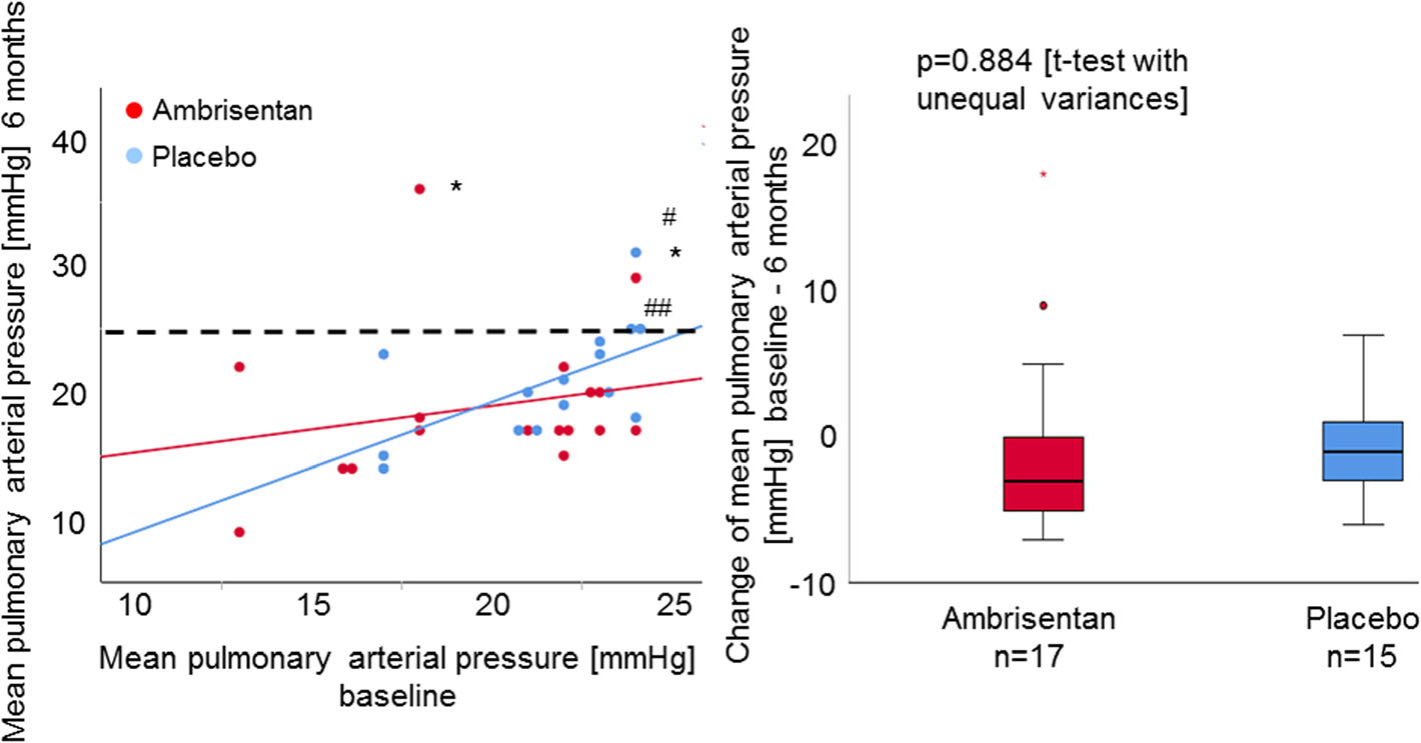
Changes of mPAP over 6 months. No patients at baseline had a resting mPAP of ≥ 25 mmHg. After 6 months, 2 patients in the ambrisentan group developed a resting mPAP of > 25 mmHg. The dotted line indicates a resting mPAP of 25 mmHg. *: Two patients in the ambrisentan group had a resting PAWP of > 15 mmHg after 6 months; they were reclassified as PH due to left heart disease. #: Three patients in the placebo group developed a resting mPAP of ≥ 25 mmHg at month 6 with a resting PAWP of ≤ 15 mmHg; thus, they were diagnosed as having SSc-APAH after 6 months. The mean change of resting mPAP over 6 months in the ambrisentan group was − 1 ± 6.4 mmHg, and that in the placebo group was − 0.73 ± 3.59 mmHg. The changes between the two groups were not significantly different (*p* = 0.884). Ambrisentan did not significantly decrease the mPAP at rest over 6 months compared to placebo

The primary endpoint, mean change of resting mPAP, was not significantly different between ambrisentan and placebo (*p* = 0.884, Table 2, Fig. 2). After 6 months, mPAP at peak exercise increased with placebo, whereas it decreased with ambrisentan, though not significantly different (*p* = 0.494).

**Table 2:** Changes of clinical parameters from baseline to 6 months

Parameter [unit]	Placebo			Ambrisentan			95% CI of difference
	Changes (*n* = 15)			Changes (*n* = 17)			
	Baseline–6 months			Baseline–6 months			
	Mean ± SD	95% CI	*n*	Mean ± SD	95% CI	*n*	
Hemodynamics at rest							
CVP [mmHg]	− 0.20 ± 2.76	(− 1.73 to 2.94)		0.82 ± 4.11	(− 1.29 to 2.94)		(− 1.54 to 3.59)
mPAP [mmHg]	− 0.73 ± 3.59	(− 2.72 to 1.26)		− 1.00 ± 6.40	(− 4.29 to 2.29)		(− 4.09 to 3.56)
PAWP [mmHg]	0.13 ± 3.20	(− 1.64 to 1.91)		1.24 ± 5.31	(− 1.50 to 3.97)		(− 2.12 to 4.32)
CO [l/min]	− 0.26 ± 1.11	(− 0.88 to 0.36)		0.58 ± 1.17	(− 0.03 to 1.18)		(0.01 to 1.66)
CI [l/min/m^2^]	− 0.31 ± 0.71	(− 0.71 to 0.08)		0.36 ± 0.66	(0.01 to 0.71)	(16)	(0.18 to 1.18)
SvO_2_ [%]	− 3.48 ± 12.26	(− 10.89 to 3.93)	(13)#	− 2.79 ± 7.56	(− 7.60 to 2.01)	(12)#	(− 7.83 to 9.20)
PVR [WU]	0.02 ± 0.76	(− 0.48 to 0.43)		− 0.59 ± 0.79	(− 1.05 to − 0.14)		(− 1.25 to − 0.17)
Hemodynamics at peak exercise							
mPAP [mmHg]	1.08 ± 7.39	(− 3.39 to 5.54)	(13)	− 0.73 ± 6.23	(− 4.18 to 2.71)	(15)	(− 7.10 to 3.48)
PAWP [mmHg]	0.85 ± 6.3	(− 2.96 to 4.65)	(13)	4.93 ± 6.52	(1.16 to 8.69)	(14)	(− 1.01 to 9.17)
CO [l/min]	− 0.05 ± 1.46	(− 0.94 to 0.83)	(13)	1.24 ± 1.47	(0.43 to 2.05)	(15)	(0.15 to 2.44)
CI [l/min/m^2^]	− 0.45 ± 1.36	(− 1.27 to 0.37)	(13)	0.70 ± 0.81	(0.25 to 1.15)	(15)	(0.29 to 2.00)
SvO_2_ [%]	− 28.35 ± 10.5	(− 35.41 to − 21.30)	(11)	− 30.05 ± 17.73	(− 42.73 to − 17.37)	(10)	(− 14.85 to 11.46)
Workload [Watt]	1.92 ± 16.01	(− 7.75 to 11.60)	(13)	5.00 ± 16.9	(− 4.36 to 14.36)	(15)	(− 9.77 to 15.93)
HR max [b/min]	6.00 ± 18.74	(− 5.32 to 17.32)	(13)	7.47 ± 12.01	(0.82 to 14.12)	(15)	(− 10.59 to 13.53)
PVR [WU]	− 0.003 ± 0.34	(− 0.21 to 0.21)	(13)	− 0.84 ± 0.48	(− 1.12 to − 0.57)	(14)	(− 1.17 to − 0.51)
TPR [mmHg min L^− 1^]	0.16 ± 0.63	(− 0.22 to 0.54)	(13)	− 0.56 ± 0.48	(− 0.83 to − 0.29)	(15)	(− 1.16 to − 0.29)
6MWD							
6MWD [m]	− 16.53 ± 77.32	(− 59.35 to 26.29)		21.53 ± 34.6	(3.74 to 39.31)		(− 4.30 to 80.43)
Borg dyspnea score	0.08 ± 1.54	(− 0.77 to 0.93)		0.62 ± 1.7	(− 0.25 to 1.50)		(− 0.63 to 1.72)
SaO_2_ after 6MWD [%]	1.30 ± 5.1	(− 2.35 to 4.95)	(10)#	2.07 ± 5.7	(− 1.22 to 5.37)	(14)	(− 3.92 to 5.46)
HR after 6MWD [/min]	6.83 ± 24.97	(− 9.04 to 22.70)	(12)#	− 1.53 ± 18.43	(− 11.74 to 8.68)	(15)	(− 25.56 to 8.83)
Quality of life SF-36							
Physical functioning	− 2.00 ± 25.20	(− 15.96 to 11.96)		− 7.65 ± 21.66	(− 18.78 to 3.49)		(− 22.56 to 11.27)
Physical role functioning	13.33 ± 38.81	(− 8.12 to 34.82)		− 10.29 ± 42.44	(− 32.12–11.53)		(− 53.14 to 5.88)
Bodily pain	− 2.47 ± 28.19	(− 18.08 to 13.15)		− 9.29 ± 23.87	(− 21.57 to 2.98)		(− 25.62 to 11.97)
General health perceptions	0.20 ± 19.39	(− 10.54 to 10.94)		− 2.71 ± 10.62	(− 8.17 to 2.75)		(− 14.01 to 8.20)
Vitality	− 5.00 ± 16.37	(− 14.06 to 4.06)		− 3.53 ± 12.34	(− 9.88 to 2.82)		(− 8.92 to 11.86)
Social role functioning	0.87 ± 27.69	(− 14.47 to 16.20)		− 2.18 ± 19.21	(− 12.05 to 7.70)		(− 20.08 to 13.99)
Emotional role functioning	8.80 ± 49.68	(− 18.71 to 36.31)		− 9.82 ± 36.76	(− 28.73 to 9.08)		(− 49.93 to 12.68)
Mental health	− 3.73 ± 11.85	(− 10.30 to 2.83)		− 4.47 ± 10.94	(− 10.10 to 1.16)		(− 8.97 to 7.49)
Physical health score	0.87 ± 16.01	(− 8.00 to 9.73)		− 6.71 ± 12.17	(− 12.97 to − 0.45)		(− 17.77 to 2.62)
Mental health score	0.27 ± 19.77	(− 10.68 to 11.22)		− 4.65 ± 9.47	(− 9.51 to 0.22)		(− 15.89 to 6.06)
mRSS	0.73 ± 2.15	(− 0.46 to 1.93)		0.24 ± 0.97	(− 0.26 to 0.73)		(− 1.68 to 0.68)
Lung function							
FVC [%]	− 1.04 ± 5.60	(− 4.15 to − 2.06)		− 3. 31 ± 5.56	(− 6.27 to − 0.34)	(16)	(− 6.37 to 1.84)
FEV1 [L]	− 0.06 ± 0.20	(− 0.17 to 0.05)		− 0.11 ± 0.21	(− 0.22 to 0.01)	(16)	(− 0.20 to 0.11)
FEV1% VC max [%]	− 0.67 ± 4.50	(− 3.16 to 1.82)		− 4.17 ± 7.12	(− 7.97 to − 0.38)	(16)	(− 7.91 to 0.91)
PEF [l/s]	−0.22 ± 1.76	(−1.12 to 0.76)		0.01 ± 1.15	(−0.61 to 0.62)	(16)	(−0.86 to 1.31)
TLC [l]	− 0.03 ± 0.36	(− 0.23 to 0.17)		− 0.06 ± 0.37	(− 0.26 to 0.14)	(16)	(− 0.30 to 0.24)
Residual volume [l]	0.05 ± 0.37	(− 0.16 to 0.25)		− 0.03 ± 0.33	(− 0.20 to 0.15)	(16)	(− 0.33 to 1.83)
DLCO [mmol/min/kPa]	− 0.45 ± 1.70	(− 1.48 to 0.57)	(13)	− 0.32 ± 1.44	(− 1.06 to 0.42)		(− 1.04 to 1.30)
DLCO % predicted	− 0.44 ± 1.84	(− 1.55 to 0.67)	(13)	1.19 ± 1.81	(0.26 to 2.12)	(17)	(25.10 to 30.04)
SaO_2_ [%]	0.15 ± 1.86	(− 0.88 to 1.18)		− 0.62 ± 1.50	(− 1.39 to 0.16)		(− 1.99 to 0.44)
PaO_2_ [mmHg]	1.69 ± 9.95	(− 3.82 to 7.21)		− 4.88 ± 7.60	(− 8.78 to − 0.97)		(− 12.92 to − 0.22)
PaCO_2_ [mmHg]	− 0.03 ± 2.63	(− 1.48 to 1.43)		− 0.65 ± 2.77	(− 2.08 to 0.77)		(− 2.58 to 1.33)
Echocardiography							
Estimated sPAP [mmHg]	− 0.93 ± 6.08	(− 4.30 to 2.43)		− 0.82 ± 4.46	(− 3.11 to 1.47)		(− 3.71 to 3.93)
RA area [cm^2^]	− 0.47 ± 4.07	(− 2.72 to 1.79)		1.65 ± 2.67	(0.28 to 3.01)		(− 0.34 to 4.57)
RV area [cm^2^]	− 0.80 ± 3.05	(− 2.49 to 0.89)		− 0.15 ± 3.46	(− 1.93 to 1.63)		(− 1.72 to 3.02)
TAPSE [cm]	− 0.19 ± 0.54	(− 0.49 to 0.11)		0.12 ± 0.41	(− 0.09 to 0.33)		(− 0.04 to 0.65)
Laboratory							
Hemoglobin [g/dl]	0.19 ± 0.68	(− 0.19 to 0.56)		− 0.59 ± 0.86	(− 1.03 to − 0.15)		(− 1.34 to − 0.21)
Hematocrit [l/l]	0.00 ± 0.02	(− 0.009 to 0.02)		− 0.01 ± 0.02	(− 0.03 to − 0.0001)		(− 0.03 to 0.00)
Platelets [100/nl]	− 0.08 ± 0.39	(− 0.29 to 0.14)		− 0.15 ± 0.37	(− 0.34 to 0.04)		(− 0.35 to 0.20)
Creatinine [mg/dl]	− 0.03 ± 0.09	(− 0.08 to 0.02)		− 0.04 ± 0.11	(− 0.09 to 0.02)		(− 0.08 to 0.07)
Potassium [mmol/l]	0.09 ± 0.40	(− 0.13 to 0.31)		− 0.06 ± 0.62	(− 0.38 to 0.26)		(− 0.53 to 0.24)
AST [U/l]	− 4.40 ± 13.94	(− 12.12 to 3.32)		3.59 ± 7.96	(− 0.51 to 7.68)		(− 0.08 to 16.06)
ALT [U/l]	− 4.93 ± 15.01	(− 13.25 to 3.38)		5.12 ± 7.83	(1.09 to 9.15)		(1.56 to 18.55)
LDH [U/l]	− 7.00 ± 27.36	(− 22.15 to 8.15)		2.82 ± 29.57	(− 12.38 to 18.03)		(− 10.84 to 30.49)
CK [U/l]	9.21 ± 40.50	(− 14.17 to 32.60)	(14)	5.53 ± 36.80	(− 13.39 to 24.45)		(− 32.10 to 24.73)
CRP [mg/l]	− 1.08 ± 3.52	(− 3.03 to 0.87)		− 2.71 ± 12.17	(− 8.97 to 3.54)		(− 8.29 to 5.03)
NTproBNP [pg/ml]	31.00 ± 85.83	(− 20.87 to 82.87)	(13)	− 15.63 ± 207.48	(− 126.18 to 94.93)	(16)	(− 172.95 to 79.70)

*SD* standard deviation, *CVP* central venous pressure, *mPAP* mean pulmonary arterial pressure, *PAWP* pulmonary capillary wedge pressure, *CO* cardiac output, *CI* cardiac index, *SvO2* venous oxygen saturation, *PVR* pulmonary vascular resistance, *WU* Wood Units, *HR* heart rate, *b* beats, *min* minute, *TPR* total pulmonary resistance, *6MWD* Six-minute walking distance, *SaO2* oxygen saturation, *HR* heart rate, *mRSS* modified Rodnan Skin Score, *FVC* forced vital capacity, *FEV1* forced expiratory volume in first second, *VC* vital capacity, *PEF* peak expiratory flow, *TLC* total lung capacity, *DLCO* diffusing capacity of the lung for carbon monoxide, *SaO2* oxygen saturation, *PaO2* partial pressure of oxygen, *PaCO2* partial pressure of carbon dioxide, *sPAP* systolic pulmonary arterial pressure, *RA* right atrial, *RV* right ventricular, *TAPSE* tricuspid annular plane systolic excursion, *AST* aspartate-aminotransferase, *ALT* alanine-aminotransferase, *LDH* lactate dehydrogenase, *CK* creatine kinase, *CRP* C-reactive protein, *NTproBNP* N-terminal pro-brain natriuretic peptide

In case of missing data, sample sizes are given in brackets

#Values with more than 20% missing data

After 6 months, 5 patients presented with resting mPAP values above ≥ 25 mmHg (placebo *n* = 3, ambrisentan *n* = 2). The 3 patients of the placebo group were classified as manifest PAH. They were aged 25, 55, and 56 years respectively, had a resting PAWP of  ≤15 mmHg both at rest and during exercise at month 6, and had shortness of breath. Their mPAP values were 24 mmHg at baseline and increased within 6 months to values ≥ 25 mmHg. In all 3 PAH patients, the disease was diagnosed at a very early stage (27 ± 3.5 mmHg), and in 2 patients, the increase of mPAP was only very mild (Fig. 2) most likely due to the short observation period. Up-front targeted PAH medication was started in these patients immediately after the second RHC was performed and the end of the study was reached.

Both PH patients in the ambrisentan group were categorized as newly developed manifest PH due to left heart diseases characterized by an increased resting PAWP of > 15 mmHg (Fig. 2). One patient was a 71-year-old female with systemic arterial hypertension. Her resting mPAP at baseline increased from 24 to 29 mmHg and resting PAWP from 11 to 18 mmHg after 6 months. Subsequent examinations including left heart catheterization showed a progression of left ventricular diastolic dysfunction. The second patient with manifest PH after 6 months was a 79-year-old female with a resting mPAP of 18 mmHg at baseline and a resting PAWP of 5 mmHg, which markedly increased to an mPAP of 36 mmHg after 6 months with a resting PAWP of 22 mmHg. Left heart catheterization revealed a coronary artery disease.

**Fig. 3:**
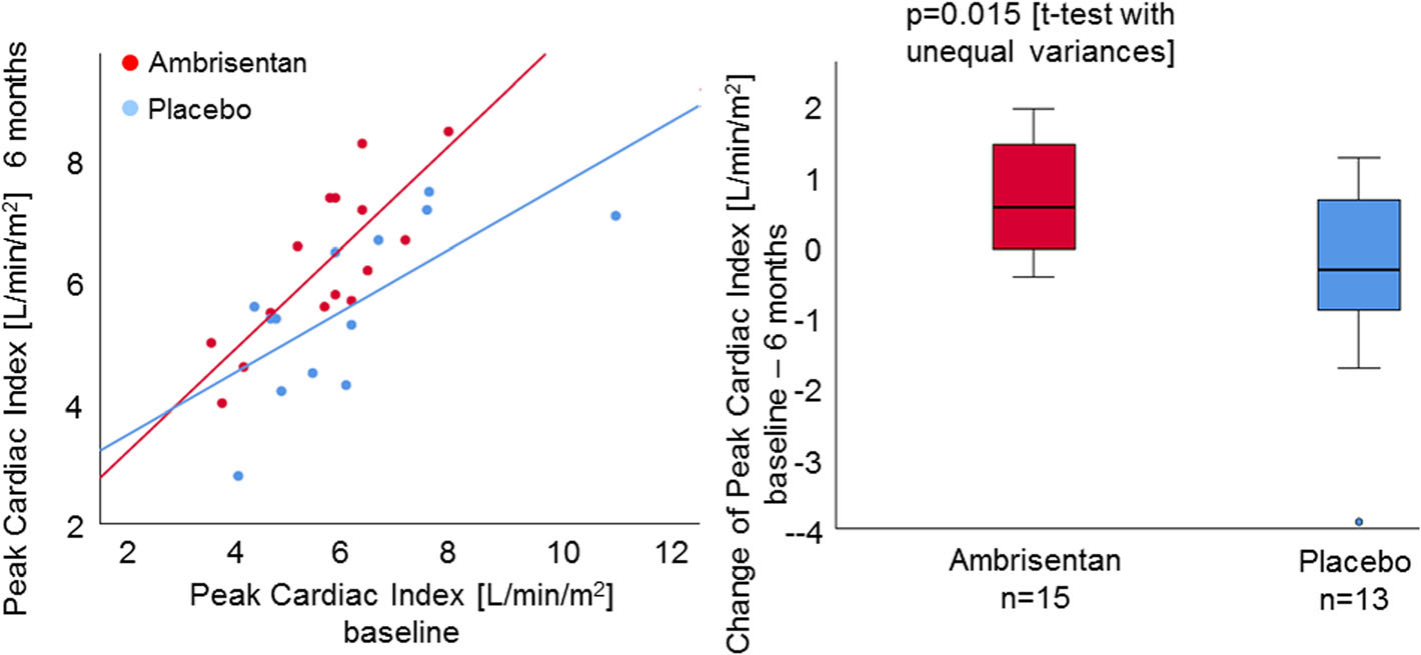
Changes of peak CI over 6 months. The mean change of CI at maximal exercise over 6 months in the ambrisentan group was 0.70 ± 0.81 L/min/m^2^ and that in the placebo group was − 0.45 ± 1.36 L/min/m^2^. Ambrisentan significantly increased the CI at maximal exercise over 6 months compared to placebo (*p* = 0.015)

**Fig. 4:**
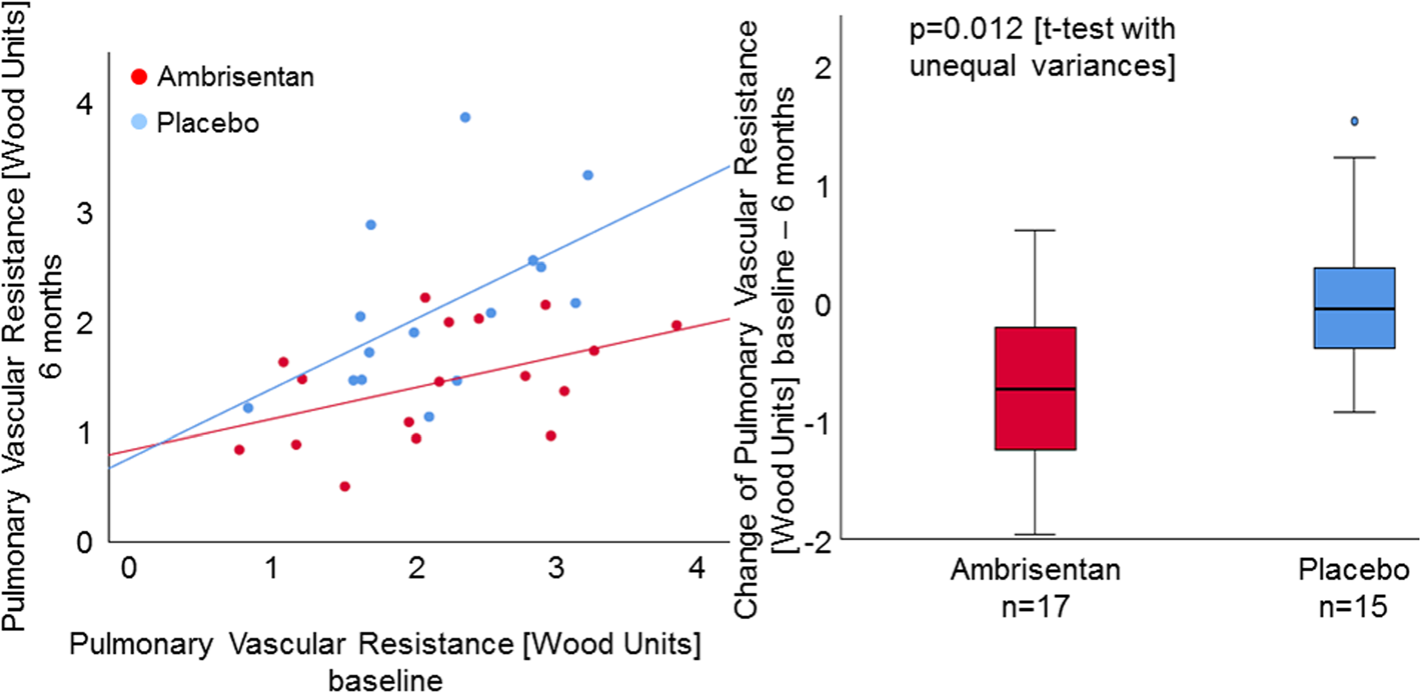
Changes of PVR at rest over 6 months. Ambrisentan patients had on average a lower PVR at 6 months compared to placebo. The mean change of PVR at rest over 6 months in the ambrisentan group was − 0.70 ± 0.78 WU and that in the placebo group was 0.01 ± 0.71 WU. Ambrisentan significantly decreased the PVR at rest over 6 months compared to placebo (*p* = 0.012)

The secondary endpoints CO and CI significantly increased in the ambrisentan group compared to those in the placebo group which tended to decrease, both at rest (*p* = 0.047, *p* = 0.010, respectively) and at peak exercise (*p* = 0.028, *p* = 0.015, respectively, Fig. 3). Furthermore, change of TPR significantly differed between groups, with a decrease of TPR in the ambrisentan group and an increase in the placebo group (*p* = 0.022, Table 2). There were no significant changes of PAWP in the ambrisentan group compared to the placebo group at rest (*p* = 0.478) or at maximal exercise (*p* = 0.111). Compared to the placebo group, PVR in the ambrisentan group significantly decreased over 6 months both at rest (Fig. 4) and at maximal exercise (*p* = 0.012, *p* < 0.0001, respectively). There was no significant difference in mPAP (*p* = 0.494), workload, and heart rate at peak exercise between the two groups.

### WHO functional class, 6-min walking test, quality of life, and skin fibrosis (Table 2)

At 6 months, 17 patients (100%) in the ambrisentan group and 13 patients (86.7%) in the placebo group were in WHO-FC II. The remaining 2 patients in the placebo group were in WHO-FC III.

After 6 months, the mean 6MWD improved by 21.53 m in the ambrisentan group and decreased by 16.53 m in the placebo group (*p* = 0.095; Table 2). No differences were detected regarding quality of life and skin fibrosis. Sensitivity analysis of patients with dcSSc did not reveal different results.

### Lung function, transthoracic echocardiography, and laboratory data (Table 2)

The changes in predicted DLCO showed a decrease in the placebo group and an increase in the ambrisentan group (95% confidence interval of the difference 0.25–3.0%, Table 2). No changes between the two groups were recorded with regard to other parameters of pulmonary function. A change in the partial pressure of oxygen (PaO_2_) was recorded, with a decrease in the ambrisentan group, whereas it slightly increased in the placebo group.

Parameters of right heart size and function did not show differences between groups.

Patients receiving ambrisentan had a not clinically relevant drop in hemoglobin concentration (− 0.59 ± 0.86 g/dl, 95% confidence interval − 1.03 to − 0.15) compared to placebo. No differences were identified regarding other laboratory parameters, including those concerning renal failure, liver damage, and NTproBNP levels.

### Safety and tolerability (Table 3)

Most of the adverse events were of mild or moderate intensity in both groups and are summarized in Table 3. Serious adverse events were more frequently reported for the placebo group (Table 3), with all events requiring hospitalization, but being resolved at the end of the study.

**Table 3:** Adverse events

Event	Placebo (*N* = 19)	Ambrisentan (*N* = 19)
	Number of patients (percent)	
Patient with at least 1 adverse event	17 (89.5)	17 (89.5)
Headache	6 (31.58)	6 (31.58)
Edema	4 (21.05)	8 (42.11)
Dizziness	6 (31.58)	0 (0)†
Diarrhea	2 (10.53)	4 (21.05)
Nausea	3 (15.79)	2 (10.53)
Paresthesia	0 (0)	4 (21.05)
Coronary artery disease	3 (15.79)	1 (5.26)
Hypotension	2 (10.53)	2 (10.53)
Epistaxis	1 (5.26)	3 (15.79)
Serious adverse events*		
Lower jaw fracture	0	1
Angina Pectoris	1	0
Coronary artery disease	1	0
Gastrointestinal infection	1	0
Lymphangitis	1	0
Raynaud	1	0

The adverse events listed here are those that occurred in at least 10% of patients (total) during the course of the study

†Statistically significant at level 0.05

*All serious adverse events fulfilled the criterion of hospitalization

## Discussion

To the best of our knowledge, EDITA is the first randomized, controlled study to assess the safety and efficacy of an early treatment with a PAH-targeted drug in patients with SSc and mildly elevated mPAP (mPAP 21–24 mmHg) and/or exercise PH in comparison with placebo. Patients did not significantly differ in the primary endpoint change of mPAP during the study. However, the secondary hemodynamic endpoints as peak TPR as well as right heart function (CO, CI), and PVR significantly improved in the ambrisentan group, both at rest and at peak exercise compared to placebo. No patients in the ambrisentan group but three patients from the placebo group progressed to SSc-associated PAH within 6 months of treatment (although for two patients the progression was only minimal). Ambrisentan was well tolerated, with a favorable safety profile.

### Effects of ambrisentan in patients with SSc and early pulmonary vasculopathy

While the primary endpoint of the study was not met, parameters of right ventricular (RV) function and PVR at rest and during exercise showed significant improvements during the study. Data from large registries have already shown the prognostic importance of CO, CI, and PVR at rest [25, 26]. These three parameters showed a significant improvement in the ambrisentan group after 6 months in our study, compared to placebo.

Besides resting values, exercise hemodynamics are able to unmask early RV dysfunction and vascular remodeling, especially in patients with SSc and mildly elevated mPAP, who usually display normal right heart function at rest [9, 27]. In our study, improvements of CO and CI at peak exercise were even more pronounced than changes observed at rest. The increase of CO and CI during exercise (also called RV output reserve) is able to provide useful information regarding prognosis of patients with pulmonary vascular diseases [28, 29].

The finding that change of TPR slope significantly differed between groups and that only patients in the placebo group presented with a manifest SSc-APAH during follow-up, whereas two patients in the ambrisentan group developed left heart disease PH, may be a hint for the beneficial effect of ambrisentan on the pulmonary vasculature, though the primary endpoint of mPAP change was not met. As two patients in the intervention group presented with left heart disease PH at the end of the study, though they did not present with significant left heart disease at baseline, treatment with ambrisentan may potentially lead to the unmasking and progression of left heart disease in patients with SSc [30]. Furthermore, pulmonary fibrosis has to be taken into account when initiating treatment with ambrisentan, as ambrisentan may lead to worsening of PaO_2_ [30], as found in our cohort, though not clinically relevant. Patients who are eligible for an early treatment with PAH-targeted drugs have therefore to be carefully selected. Furthermore, hemoglobin as well as hepatic values have to be thoroughly monitored. Further, larger-scaled studies are needed to investigate the effect of early PAH-targeted treatment in SSc patients with mildly elevated mPAP and/or exercise PH and its impact on the pulmonary vascular system and right heart function.

### Comparison with previous reports on treatment with PAH-targeted drugs in patients with SSc and early pulmonary vasculopathy

Two uncontrolled small open-label reports were previously performed, one with ambrisentan [15] and one with bosentan [16]. Our data are in line with those published by Saggar et al. on 12 patients with exercise induced PH < 25 mmHg at rest and > 30 mmHg at peak exercise receiving ambrisentan treatment for 24 weeks. After 24 weeks of treatment, the authors did not find a significant decrease of resting mPAP (*p* = 0.65) but a remarkable improvement of CO at rest and during exercise (*p* = 0.01 and *p* = 0.006 respectively) and of PVR during exercise (*p* = 0.003).

Kovacs et al. assessed in a retrospective uncontrolled study 10 patients with mildly elevated mPAP (21–24 mmHg) receiving bosentan for 24 weeks. Apart from an improvement in right heart function (CO at rest *p* = 0.05), they reported a significant reduction of mPAP at rest and peak exercise (*p* = 0.03 and *p* = 0.02, respectively) after 24 weeks of bosentan treatment [16]. Congruently with the results of Kovacs and Saggar, we did not find significant improvements in 6MWD, QoL, and WHO-FC. Since more than 70% of our cohort had 6MWD > 400 m and WHO-FC II at baseline, our findings are concordant with the results of a post hoc analysis of the ambrisentan in PAH, randomized, double-blind, placebo-controlled, multicenter efficacy study 1 and 2 (ARIES)-1 and ARIES-2 studies, which reported that ambrisentan had a greater effect on 6MWD and WHO-FC in patients with more severe PAH [31].

### Clinical implication and safety

Pulmonary vascular abnormalities in PVR have been shown to be already remarkable even in patients with only mild symptoms [32] and may precede an overt disease [33]. The improvement in PVR observed in our cohort might indicate that active treatment with ambrisentan is able to prevent the progressive vascular remodeling in SSc patients with an early form of pulmonary vasculopathy.

Ambrisentan treatment has shown a good safety profile, which is in line with the current analysis of safety and tolerability [34, 35]. Undesirable effects of ambrisentan recorded in our study were a clinically not relevant drop of hemoglobin concentration and PaO_2_. The reduction of hemoglobin found in our study may be due to a class effect of all endothelin receptor antagonists, and the most probable cause might be fluid retention [36]. A non-dose-dependent change in hemoglobin values was also found in the first 12 weeks of the ARIES-1 and ARIES-2 studies, with stabilization during the subsequent 24 weeks [11]. Long-term extension of the ARIES studies confirmed that hemoglobin levels tended to stabilize over time [37].

### Limitations

Our study may have been biased due to the inclusion criteria of including patients with mildly elevated mPAP both at rest and during exercise. The distribution of these entities may have influenced the results, though it did not differ between the two groups. As there is growing evidence that both states with mildly elevated pressures at rest as well as during exercise bear the risk of developing manifest pulmonary vascular disease, we included both phenotypes into the study. Furthermore, a longer study period of more than 6 months may have helped to interpret the results, as longer time intervals could have led to more distinct changes in pulmonary hemodynamics. As two patients of the placebo group with manifest PAH at follow-up increased only minimal, the changes are too small to draw definite conclusions. Larger-scaled studies with longer observation periods are needed to further investigate this issue.

Among our study cohort, two patients developed left heart disease PH during the study period, which was based on PAWP at rest and during exercise. While it would be preferable to clearly distinguish the effects of targeted treatment on the pulmonary vasculature, it is hardly possible to exclude interfering diseases such as left heart and lung disease. Patients in our study were only enrolled in the absence of significant left heart or lung disease at baseline to limit determining factors of mPAP elevation and development of manifest PH. To further characterize patients with PH due to left heart disease, the assessment of the diastolic pressure gradient and echocardiographic parameters of diastolic dysfunction would have been desirable. The higher frequency of patients with lcSSc and shorter duration of SSc in the ambrisentan group may have influenced the results, though sensitivity analysis of patients with dcSSc did not show different results. “Development of PAH” as a primary endpoint would have required a large sample size, which would not have been feasible in a single-center study.

## Conclusions

Although the primary endpoint was not met (change in mPAP over 6 months), ambrisentan was associated with significant improvements of secondary endpoints, such as peak TPR, PVR, CO, and CI both at rest and at peak exercise. Ambrisentan showed good safety and tolerability in this patient cohort. The potential risks of ambrisentan of unmasking left heart disease, drop of hemoglobin, and PaO_2_ have to be taken into account before treatment initiation and thoroughly monitored during treatment. All SSc patients included in this study had symptoms such as shortness of breath with a WHO-FC II-III, some of them revealed already an impaired RV function at rest and during exercise. Thus, these patients had already a cardio-pulmonary disease and further treatment studies are mandatory. Determinants of treatment response as well as development of comorbidities should also be a focus of future studies. The results of this study will be helpful to design future trials.

## Data Availability

The data is available from the corresponding author upon reasonable request.

## Abbreviations

6MWD: 6-min walking distance
CI: Cardiac index
CO: Cardiac output
dcSSc: Diffuse cutaneous systemic sclerosis
DLCO: Diffusion capacity of the lung
ECG: Electrocardiogram
FVC: Forced vital capacity
HRCT: High-resolution computed tomography
lcSSc: Limited cutaneous systemic sclerosis
mPAP: Mean pulmonary arterial pressure
mRSS: Modified Rodnan skin score
NTproBNP: N-terminal fraction of the pro-brain natriuretic peptide
PAH: Pulmonary arterial hypertension
PAWP: Pulmonary arterial wedge pressure
PH: Pulmonary hypertension
PVR: Pulmonary vascular resistance
QoL: Quality of life
RHC: Right heart catheterization
RV: Right ventricular
SSc: Systemic sclerosis
SvO_2_: Venous oxygen saturation
TPG: Transpulmonary gradient
TPR: Total pulmonary resistance
TnT: Troponin T
WHO-FC: World Health Organization functional class
